# Prevalence of canine cranial cruciate ligament rupture and prognosis depending on tibial plateau angle: A retrospective study

**DOI:** 10.5455/javar.2024.k812

**Published:** 2024-09-29

**Authors:** Beom-Seok Seo, Chul Park, Md. Mahbubur Rahman, Inseong Jeong, Namsoo Kim

**Affiliations:** 1 Royal Dog and Cat Medical Center,Seoul, Republic of Korea; 2 Department of Veterinary Surgery, College of Veterinary Medicine, Jeonbuk National University, Jeonju, Republic of Korea; 3 Gachon Pain Center and Department of Physiology, College of Medicine, Gachon University, Incheon, Republic of Korea; 4 Department of Veterinary Surgery, Royal Animal Medical Center, Seoul, Republic of Korea; † These authors contributed equally to this work.

**Keywords:** Cranial cruciate ligament rupture, small breeds dogs, Tibial plateau angle, Tibial plateau leveling osteotomy

## Abstract

**Objective:**

This study aimed to evaluate the prevalence of cranial cruciate ligament rupture (RCCL) and the efficacy of tibial plateau angle (TPA) as a prognostic indicator in small breed dogs diagnosed with RCCL.

**Materials and Methods:**

For this study, 53 dogs with RCCL were selected based on their medical data. The Tibial plateau angle (TPA) was calculated by analyzing the radiographic images of the stifle joint region. The dogs were categorized based on their body weight, sex, age, breed, and RCCL. Subsequently, clinical outcomes of dogs were evaluated based on their pre- and postoperative TPAs.

**Results:**

The prevalence of RCCL was higher in neutered dogs, especially those neutered before 6 months of age. The preoperative TPAs of all dogs diagnosed with RCCL were lowered with TPLO. Preoperative walking time in the higher pre-operative TPA (>25°) group (13.58 ± 1.53 days) was significantly (*p *< 0.05) longer than the lower pre-operative TPA (≤25°) group (10.09 ± 0.84). The lower pre-operative TPA (≤ 25°) group showed better prognoses without any more complications than the higher pre-operative TPA (> 25°) group. Furthermore, post-operative walking time in the higher post-operative TPA dogs (>10°) group (18.08 ± 2.22 days) was significantly (*p* < 0.05) longer than the lower post-operative TPA dogs (≤10°) group (10.20 ± 0.90 days). Likewise, the TPA (≤10°) group showed better prognoses with lowered complications.

**Conclusion:**

Therefore, pre- and post-operative TPA plays an important prognostic factor, and post-operative TPA should be kept at<10° to get better clinical outcomes.

## Introduction

The major cause of cranial cruciate ligament rupture (RCCL) in canines is osteoarthritis, progressive stifle joint degeneration, and hind limb lameness [1]. Prior research has provided prevalence figures ranging from 1.2% to 2.6% for CCL disorders in dogs. It has also found several risk variables associated with this condition, such as breed, sex, neutering status, age, and body weight (BW) [1–3]. The majority of these articles are about large breeds. Nevertheless, the dog breeds predominantly adopted by South Koreans are small breeds [1,4]. Therefore, the prevalence of RCCL in the Republic of Korea needs to be described.

There are many operative techniques (over 60) reported to treat RCCL [5,6], and currently, the tibial plateau leveling osteotomy (TPLO) or tibial tuberosity advancement (TTA) is frequently used. The objective of these techniques is to neutralize the cranial tibial thrust, thereby achieving stifle joint stability while weight-bearing [7]. One of the most commonly conducted of these procedures is the TPLO, which was first described in 1993 [8,9]. TPLO is more appropriate to neutralize cranial tibial thrust and is indicated especially when the tibial plateau slope is excessive [9,10]. In this study, TPLO surgery was performed to correct RCCL.

The preoperative [11] and postoperative Tibial plateau angle (TPA) [11,12] affect the clinical outcome after TPLO surgery. The objective of attaining a postoperative TPA of 5° is the standard for getting better clinical outcomes [13]. However, it is difficult to obtain this range every time. In addition, contrasting reports have been published, such as no significant difference in post-operative limb function reported for a post-operative TPA of 0°–14° [14] and superior clinical outcomes in TPAs ≤ 14° compared with those with post-operative TPAs > 14° [11]. The dogs included in both studies were also from large breeds. However, in Asia, including the Republic of Korea, small breeds are the most common.

The occurrence of RCCL and clinical outcomes depending on TPA in small breeds remain unclear. Hence, the main objective of this study was to explore the effects of pre-operative (by dividing the dogs into a TPA > 25° group and TPA ≤ 25° group) and post-operative TPA (TPA > 10° group and TPA ≤ 10° group) in TPLO as a prognostic factor in RCCL dogs. In addition, the prevalence of RCCL was also investigated.

## Materials and Methods

### Ethical approval

The expert veterinary surgeon from the Royal Animal Medical Center diagnosed the clinical cases and recommended surgical treatment during a team meeting. The committee on the care of animal resources approved all the procedures used in this study (Approval number: RAMC IACUC 20-RS-08).

### Criteria for selecting a case

The selection of cases was conducted by a thorough examination of medical records obtained from three veterinary centers: Royal Animal Medical Center, Royal Dog and Cat Medical Center, and Seoul Animal Medical Center. The records of the three institutions were examined from January 2017 to October 2019, specifically focusing on dogs that had undergone stifle joint radiography, either showing normal conditions or with an RCCL. Each dog was evaluated for pelvic limb lameness resulting from a suspected partial or full RCCL.

The RCCL was initially diagnosed by the examination of various clinical signs, including the cranial drawer sign, tibia push, joint effusion, medial buttress, and meniscal click. Finally confirmed by consistent radiographic evidence of stifle joints and ligaments, the presence of a partial or complete RCCL in surgical procedures was considered for study inclusion.

### Clinical history

Data collected from medical records included the patient’s age, weight, gender, the activity related to the initial lameness, affected limb, the period of the lameness, analgesic treatment, the radiographic findings before and after the surgery, and the TPA measurement, and finally recorded on standardized survey sheets. TPA underwent evaluation by a medical team consisting of imaging diagnostic experts and surgeons. The study exclusively comprised dogs that had thorough medical records and scheduled regular follow-up visits. There were a total of 15 different breeds of dogs identified with RCCL (*n =* 53), including Cocker Spaniel, Maltese, Mix Dog, Yorkshire Terrier, Poodle, Welshcock, Bichon Frise, White Terrier, Golden Retriever, Jack Russell Terrier, Mini Pin, Chihuahua, Shitzu, Beagle, and Spitz. Among them were a total of 36 male dogs (non-castrated male 3 and 33 castrated males) and 17 female dogs (spayed female 13 dogs and non-spayed female 4).

### Diagnosis of RCCL

For mediolateral radiographs, the dogs were placed in lateral recumbence, with the tarsus and stifle joints flexed at a 90° angle and the limb aligned parallel to the digital image-capturing device. Radiography was performed using the Titan 2,000 machine manufactured by COMED Medical Systems Co. Ltd. in Seoul, Korea. The radiographs were then analyzed for all animals. The X-ray beam was positioned precisely over the central part of the tibia and adjusted to include the ankle joint, the whole length of the tibia, and the lower part of the thigh bone. The process of aligning the rotation correctly was accomplished by placing the femoral condyles and talar trochlea [1]. The caudocranial radiographs were acquired by placing the dog in a supine position with the limbs elongated towards the tail, in alignment with the digital imaging scanner.

The beam of X-rays was accurately placed along the proximal tibia and fixed to align with the collimation employed to take the mediolateral radiographs. The fabellae on the femoral condyles were aligned to achieve precise rotational alignment, and the medial part of the calcaneus was aligned toward the distal intermediate ridge of the shaft of the tibia [1]. When there was a difference in rotational alignment, the priority was to align the fabellae with the femoral condyle. Once there was a discrepancy in the rotational alignment, the main concern was to synchronize the fabellae with the femoral condyle.

**Figure 1.**
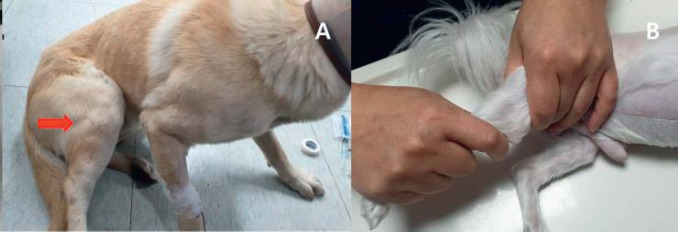
Preliminary diagnosis of cranial cruiciate ligament ruptures in dogs by Sit’s Test (A) and cranial drawer sign test (B).

### The quantification of TPA

Three individual blind examiners measured each leg for TPA (Fig. 1). The TPAs before and after the surgery were measured using the previously reported method [1]. The cranial tibial plateau landmark was determined to be the uppermost part of the medial tibial plateau, whereas the caudal landmark was defined as the lowermost part of the medial tibial plateau. A line was drawn from the highest point to the lowest point of the tibial plateau in order to measure the slope of the tibial plateau (referred to as line a). A secondary line, labeled as line b, was traced from the midpoint of the intercondylar estates to the midpoint of the talus. Line b defines the longest axis of the tibia across the sagittal axis. A third line, labeled as line c, was traced transverse to the longitudinal direction of the tibia near the junction of lines a and b intersected. The TPA was calculated by measuring the angle generated by the intersection of lines a and c. The statistical analysis used the mean of three measurements that were measured by three examiners (Fig. 1).

### Surgical procedure

The dogs received intravenous (i.v.) 0.9% normal saline at a rate of 10 ml per kilogram per hour for surgical preparation. They were also given Cephradine intravenously (30 mg/kg) twice daily, tramadol intravenously (2 mg/kg) three times daily, and either 1 mg/kg of dexamethasone intramuscularly or 15–30 mg/kg of methyl prednisolone sodium succinate intramuscularly.

The patient was anesthetized generally by intravenous propofol injection at a slow rate of 6–8 mg/kg (Provive 1%, Myungmoon Pharm. Co., Ltd., Seoul, Republic of Korea), and sevoflurane inhalant (1%–5%; Abbott Korea Ltd., Seoul, Republic of Korea) was used to maintain anesthesia. During the surgical procedure, the patient’s oxygen intake and positive pressure were regulated using an anesthetic machine (PAIEON, J & TEC, Goyang-Si, Gyeonggi-do, Republic of Korea). The patient’s electrocardiogram and CO2 partial pressure were continuously monitored, as previously reported [1,4]. Injectable opioids were delivered to all animals at intervals of 4–6 h, while antibiotics were given every 8 h during the initial 24 hours after the operation. The oral medications provided were Tramadol at a dosage of 2–4 mg/kg every 8–12 h, cephalexin at a dosage of 22 mg/kg every 12 h, and non-steroidal anti-inflammatory drugs as determined by the doctor for a period of 7–14 days after the operation.

**Figure 2.**
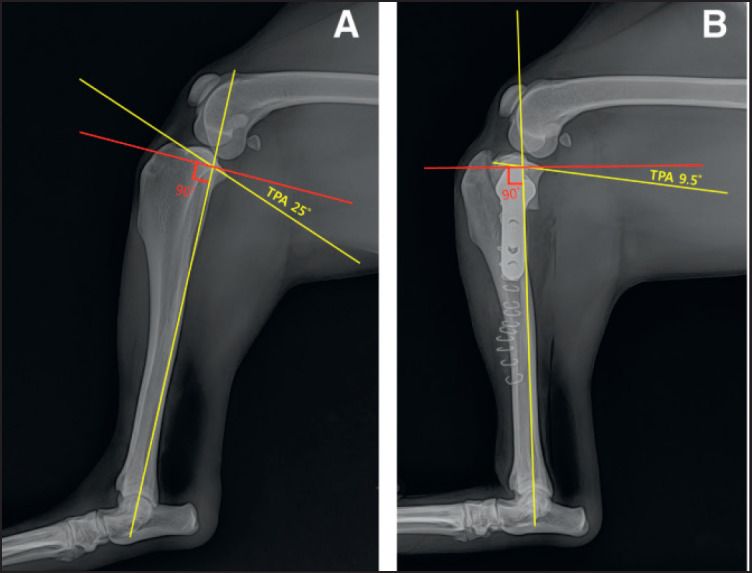
Measurement of TPA from radiographic representation of tibial plateau angle in normal and RCCL dog Preoperative (A) and post-operative (B) radiographic evaluation of tibial plateau angle and confirmation of TPLO.

Following the shaving of hair from the entire stifle joint area, a sterile preparation was carried out, involving clipping and draping of the region. Ventral recumbency was the position of the animal. A TPLO procedure was performed after dissecting the subcutaneous tissue. The tibial portion of the stifle joint was uncovered, and TPLO surgery was conducted as previously explained [14]. Briefly, the stifle joint was accessed by creating a surgical incision in the skin on the center of the stifle joint. The tibial component of the joint was then exposed, and the tibial blade was used to cut this part. Then, a tibial fracture was made for tibial movement. The distance between the tibial markings was checked carefully. Temporary pins were mounted for tibial movement. Then, movement of the tibial bone fragment was performed. Next, fixation of the plate and screw was performed. The length and types of plate and number of screws varied depending on patient to patient and pathological condition. After the TPLO procedure, a tibial compression test was reassessed intraoperatively. Finally, the surgical site was closed routinely (Fig. 2).

### Clinical outcome

Outcomes were defined as excellent: when dogs walked normally within 2 weeks after TPLO surgery; good: when dogs walked normally within 1 month after TPLO surgery; fair: when post-operative walking status was unchanged from the pre-operative conditions; complication: when post-operative walking status was worsened from the pre-operative condition at discharge, or further clinical deficits arise throughout the course of treatment that were not initially present at admission, or in the event of mortality.

### Statistical analysis

The data were analyzed using a Bonferroni post hoc test after conducting either a one-way analysis of variance (ANOVA), a two-way ANOVA, or a paired sample *t*-test. The study was performed using Prism 5.03 software from Graph Pad Software Inc., located in San Diego, CA. The data were reported as the mean ± standard error of the mean (SEM) or proportion. Statistical significance was determined when *p* < 0.05. The percentage of data was analyzed using a two-sample proportion test with Minitab software (version 16.1) to determine if there were any significant differences between the two groups.

## Results

### Data clinical outcome depending on pre-operative TPA

**Figure 3.**
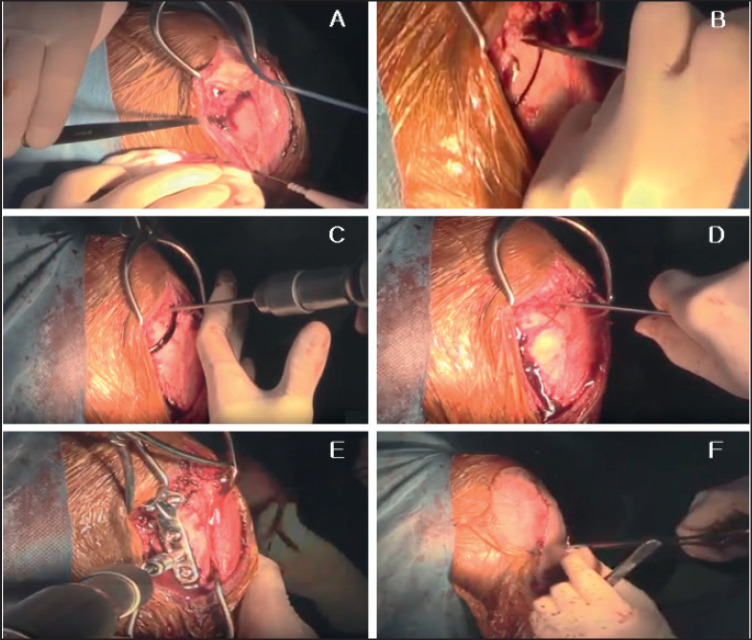
Intraoperative images of TPLO. (A) Subcutaneous tissue incision, tibial exposing, and tibial blade approaching; (B) Cutting of tibial part and fracture were making for tibial movement; (C) Temporary pin mount for tibial movement; (D) Tibial bone fragment movement; (E) Fixation of plate and screw; (E) Finally closing the incision by suturing after completion all procedures.

**Figure 4.**
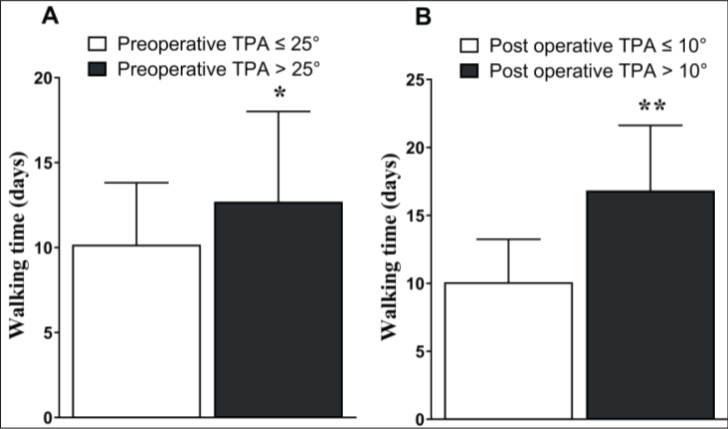
Comparison of walking time between two types of preoperative TPA and post-operative TPA.

The pre-operative TPAs of the 22 dogs were less than 25° (≤25°), and 31 dogs were greater than 25° (>25°). The average preoperative TPA for the TPA ≤ 25° group was 22.17° ± 0.60 and was 29.92° ± 0.52 (significantly higher) for the TPA > 25° group. Post-operative walking time in the pre-operative TPA > 25° group (13.58° ± 1.53° days) was significantly (*p* < 0.05) longer than lower than in the TPA ≤ 25° group (10.09° ± 0.84) (Fig. 3 and Table 1). Furthermore, in the TPA ≤ 25° group, 100% of dogs had excellent and good prognoses, while 17/22 (77.27%) of dogs had an excellent outcome, and 5/22 (22.73%) dogs had a good outcome in the TPA > 25° group. There were no dogs in the fair and complication groups (Fig. 4 and Table 1).

### Clinical outcome depending on post-operative TPA

**Figure 5.**
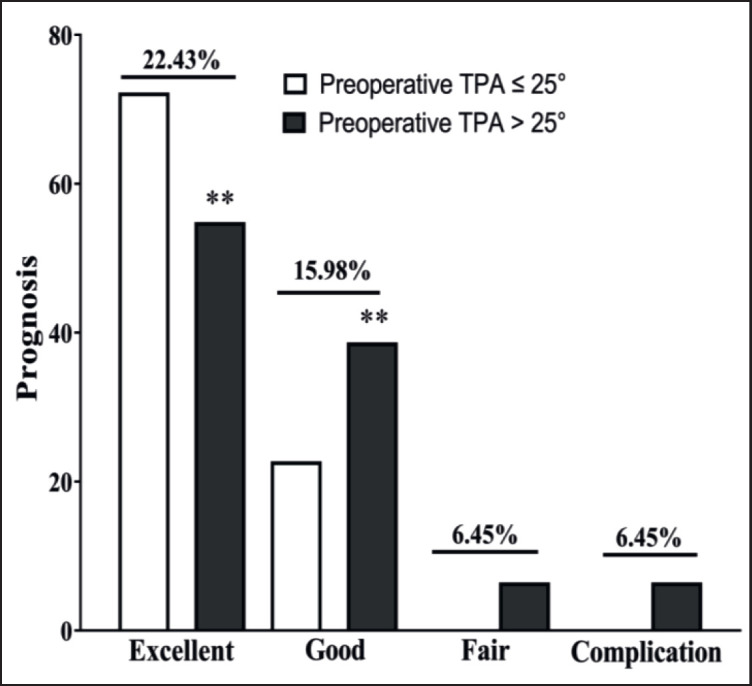
Prognosis depending on preoperative TPA. The bars above columns in Figures indicate differences between the two groups.

The post-operative TPAs of 40 dogs were less than 10° (≤10°), and 13 dogs were greater than 10° (>10°). The average of the post-operative TPA ≤ 10° group was 5.64 ± 0.43 and the TPA > 25° group was 14.40 ± 1.11 (significantly higher). Postoperative walking time in the postoperative TPA > 10° group (18.08 ± 2.22 days) was significantly (*p* < 0.05) longer than the postoperative TPA ≤ 10° group (10.20 ± 0.90 days). Furthermore, in the TPA > 10° group, 29/40 (72.50%) dogs had an excellent outcome, 10/40 (25.00%) dogs had a good outcome, 1/40 (2.50%) dogs had a fair outcome, and 1/40 dogs (2.50%) had a complication outcome (Fig. 5 and Table 1).

### Clinical outcomes depending on breed-specific preoperative and postoperative TPA

Clinical outcomes were compared in breeds specifically with low and high pre-operative TPAs to find out the effect of TPA on clinical outcomes. In most of the cases, dogs with lower TPAs walked earlier than dogs with higher preoperative TPAs. The Cocker spaniel dogs with pre-operative TPAs ≤ 25° walked sooner (average 10.67 days) than the Cocker spaniel dogs with pre-operative TPAs > 25° (average 24.6 days). Similarly, Maltese walking times were 5.50 days and 14.00 days, respectively; in Poodles 8.33 days and 12.00 days, respectively (Table 2).

Clinical outcomes were also compared in breeds, specifically with low and high postoperative TPAs, to find out the effect of TPA on clinical outcomes. In all cases, the dogs with lower TPAs walk sooner than dogs with higher postoperative TPAs. The Cocker spaniel dogs with post-operative TPAs ≤ 10° walked sooner (average 16.60 days) than the Cocker spaniel dogs with post-operative TPAs > 10° (average 24.00 days). Similarly, Maltese walking times were 8.60 days and 17.33 days, respectively; in mixed dogs, 7.60 days and 17.00 days; and in poodles, 6.50 days and 17.50 days, respectively (Table 3).

### Occurrence of RCCL depending on sex, breed, and age

The occurrence of RCCL in males was 67.92% (36/53), which was markedly greater (*p* < 0.01) than in females 32.08% (17/53). The occurrence of neutered dogs was 86.79% (46/53), which was markedly greater (*p* < 0.001) than intact dogs 13.21% (7/53) (Table 4). The occurrence in intact males was significantly lower than in castrated males. The occurrence in intact females was significantly lower than in spayed females. The occurrence of neutered before 6 months’ dogs was significantly higher than neutered after 6 months’ dogs (Table 5). The pre-operative TPA values of neutered dogs were markedly greater than those of intact canines. Comparable trends were noted in male dogs who had been castrated and female canines who had been spayed. The pre-operative TPA levels in dogs that had been neutered before 6 months were notably greater than those in dogs neutered after 6 months (Table 5).

**Table 1.** Prognosis of RCCL rupture diagnosed dogs depending on pre and postoperative tibial plateau angle.

Pre and post operative TPA° status	No of Animal	Preoperrative TPA°	Postoperrative TPA°	Difference of TPA°	Walking time (days)	Prognosis			
						Excellent	Good	Fair	Complication
Preoperrative TPA**°≤**25**°**	22	22.17 ± 0.60	7.35 ± 0.89	14.83 ± 1.05	10.09 ± 0.84	17 (77.27%)	5 (22.73%)	-	-
Preoperrative TPA**°**> 25**°**	31	29.92 ± 0.52***	8.20 ± 0.98	21.72 ± 0.93	13.58 ± 1.53*	17 (54.84%)	12 (38.71%)	2 (6.45%)	2 (6.45%)
Postoperrative TPA**°≤**10**°**	40	26.16 ± 0.75	5.64 ± 0.43	20.51 ± 0.86	10.2 ± 0.90	29 (72.50%)	10 (25%)	1 (2.50%)	1 (2.50%)
Postoperrative TPA**°**> 10**°**	13	28.40 ± 1.33	14.40 ± 1.11***	13.77 ± 1.44	18.08 ± 2.22**	5 (38.46%)	7 (53.85%)	1 (7.69%)	1 (7.70%)

Data presented as mean **±** SEM values. *p < 0.05, ** p < 0.01, *** p < 0.001 analyzed by follow-up paired sample t-test versus Preoperrative TPA°≤25 group or Preoperrative TPA°≤10 group.

The average age of the RCCL-diagnosed dogs was 7.04 ± 0.41 years. The impact of age on the incidence of TPA was assessed by categorizing dogs into four groups based on their age: less than 3 years, 3–5 years, 5–10 years, and over 10 years. The occurrence was 3.77%, 22.64%, 52.83%, and 20.75%, respectively (Fig. 4). The occurrences of the 3–5 years (*p* < 0.05), 5–10 years (*p* < 0.01), and over 10 years’ groups (*p* < 0.05) were significantly higher than the less than 3 years-aged group. Nevertheless, there were no notable disparities in the pre-operative TPAs and post-operative TPAs among the various groups, as indicated in Table 6.

## Discussion

According to these reports, cranial tibial thrust is directly associated with the TPA [9,10]. Thus, a larger tibial plateau angle (TPA) leads to an increase in cranial tibial thrust, thereby raising the risk of RCCL. The average TPA of RCCL-diagnosed small breed dogs was around 28°, which is higher than normal dogs (around 20°). Therefore, higher TPA could be considered a good parameter for preclinical diagnosis of RCCL and also a predictor of RCCL.

In this study, we found that if the pre-operative TPA was ≤ 25°, then the normal walking time after surgery was significantly shorter, without any complications, as compared with pre-operative TPA > 25°. It might be the extensive proximal tibial plateau rotation required to achieve a 5° TPA in the TPA > 25 groups, which may give extra stress and require additional time to heal. Talaat et al. [12] demonstrated that extensive tibial plateau rotation for correction of RCCL in a higher preoperative TPA group is associated with a higher complication rate.

Furthermore, the post-operative walking time of post-operative TPA ≤ 10° dogs was significantly shorter with fewer complications as compared to the post-operative TPA > 10° group. The better clinical outcomes in the TPA > 10° group may be explained by the lower tibial thrust. Thus, lower postoperative TPA (TPA > 10°) could potentially be obtained to reduce tibial thrust [13] and get better results as well as reduce complications in dogs with RCCL.

**Table 2.** Breed-specific prognosis of RCCL rupture diagnosed dogs depending on preoperative tibial plateau angle.

Breeds	No of animal with preoperrative TPA ≤25°	Average walking time (days)	No of animal with preoperrative TPA > 25°	Average walking time (days)
Cocker Spaniel	3	10.67	5	24.60
Maltese	2	5.50	6	14.00
Mix dog	1	18.00	6	9.00
Poodle	3	8.33	3	12.00
Yorkshire Terrier	2	14.00	4	12.50
Welsh corgi	3	10.33	2	6.00
Bichon Frize	1	9.00	2	13.00
Golden Retriever	2	11.50		
White Terrier	1	7.00	1	17.00
Jack Russel Terrier	1		1	12.00
Mini pin	1	7.00		
Chihuahua	1		1	7.00
Shitzu	1	7.00		
Beagle	1	15.00		
Spitz	1	9.00		

**Table 3.** Breed-specific prognosis of RCCL rupture diagnosed dogs depending on postoperative tibial plateau angle.

Breeds	No of animal with Preoperrative TPA ≤25°	Average walking time (days)	No of animal with Preoperrative TPA > 25°	Average walking time (days)
Cocker Spaniel	5	16.60	3	24.00
Maltese	5	8.60	3	17.33
Mix dog	5	7.60	2	17.00
Poodle	4	6.50	2	17.50
Yorkshire Terrier	5	11.60	1	20.00
Welsh corgi	5	8.60		
Bichon Frize	3	11.67		
Golden Retriever	1	8.60	1	13.00
White Terrier	2	11.67		
Jack Russel Terrier	1	10.00		
Mini pin	1	12.00		
Chihuahua	1	12.00		
Shitzu	1	7.00		
Beagle	1	7.00		
Spitz	1	15.00	1	9.00

The objective of this study was to ascertain the prevalence and potential factors (such as breed, BW, sex, age, and other variables) associated with RCCL in canines. The prevalence of RCCL in male dogs was 67.92%, a statistically significant increase compared to the 32.08% prevalence in female dogs. These results also conflict with previous studies, where female canines have been observed to have a higher incidence of canine RCCL [2,3,6,15]. These results might be for higher populations of male dogs, due to more popularity over female dogs in South Korea.

The occurrence of RCCL in neutered dogs and TPA was significantly higher than in non-neutered dogs. Likely, castrated males were higher than non-castrated dogs; spayed females were higher than non-spayed females. The exact cause of this discovery is yet uncertain; however, it may be linked to obesity and hormone dysregulation in neutered dogs [6]. To the best of our knowledge, we first found that higher TPA in neutered dogs was associated with RCCL. One study indicated that neutering dogs before 6 months of age could increase their susceptibility to RCCL [11]. Nevertheless, the occurrence of neutering before 6 months (20.75%), which was significantly lower than that after 6 months (79.25%), might be because of higher populations of the latter group and because of the effects of degenerative diseases (such as patella and arthritis). Nonetheless, our results indicated that the dogs that were neutered before 6 months were at higher risk of induced RCCL, as pre-operative TPA was significantly higher than in the neutered after 6 months group.

**Table 4.** Breed dependence occurrence of cranial cruciate ligament rupture.

SL	Breeds	No of dog (*n = *53)	Occurrence
1	Cocker Spaniel	8	15.09% (8/53)
2	Maltese	8	15.09% (8/53)
3	Mix dog	7	13.21% (7/53)
4	Poodle	6	11.32% (6/53)
5	Yorkshire terrier	6	11.32% (6/53)
6	Welsh corgi	5	9.43% (5/53)
7	Bichon Frise	3	5.66% (3/53)
8	Golden Retriever	2	3.77% (2/53)
9	White Terrier	2	3.77% (2/53)
10	Jack Russell Terrier	1	1.89% (1/53)
11	Mini pin	1	1.89% (1/53)
12	Chihuahua	1	1.89% (1/53)
13	Shitzu	1	1.89% (1/53)
14	Beagle	1	1.89% (1/53)
15	Spitz	1	1.89% (1/53)

In this study, we found that height occurrence in Cocker Spaniels was 15.09% Maltese breed, followed by 13.21% mix dogs, 11.32% Poodle, 11.32% Yorkshire terrier, and 9.43%. These results were inconsistent with the previous report by Taylor-Brown et al. [2]. From England, they reported the lowest prevalence of RCCL in Cocker Spaniels, and higher reports were mainly in large breeds such as crossbred dogs, Rottweilers, West Highlands, White Terriers, Golden Retrievers, and so on. Previous research conducted on 1.25 million dogs in the United States, primarily from a referral population, over a span of 40 years, revealed that the Newfoundland, Labrador retriever, Rottweiler, Boxer, and Bulldog were the five breeds most frequently affected by RCCL [16]. Follow-up investigations have verified a higher occurrence of RCCL in larger dog breeds [17]. These all-Western reports are inconsistent with our results and may vary throughout different regions, depending on the prevalence of different breeds. Small breeds are more popular in the Republic of Korea, while large breeds are more common abroad.

Higher BW results in heightened forces exerted on the limbs, leading to the consequent load on ligaments and hastening the degenerative process of the CCL [6,18]. In large breeds, Taylor-Brown et al. [2] found a notable association between higher BW in dogs and the incidence of RCCL. Interestingly, in this study, it was also found that the occurrence of RCCL in less than 10 kg BW, 10–25 kg BW, and over 25 kg BW groups were 58.49%, 35.85%, and 5.66%, respectively. The lowest BW group showed the highest occurrence in this study. The exact etiology is currently uncertain, necessitating additional inquiry to ascertain the comparative importance of BW and obesity in the pathogenesis of CCL disease. Furthermore, it is imperative to account for the same breed, age, sex, and neutering status to draw accurate conclusions.

**Table 5.** Gender and neutering dependence occurrence of RCCL rupture.

Sex and neutering	No of dogs	Total no	Occurrence	Preoperative TPA
Male	36	53	67.92% (36/53)	26.98 ± 0.88
Female	17		32.08% (17/53)**	26.13 ± 0.89
				
Intact	7	53	13.21% (7/53)	24.81 ± 1.82
Neutered	46		86.79% (46/53)***	27.00 ± 0.70*
				
Intact male	3	36	8.33% (3/36)	24.68 ± 3.91
Castrated male	33		91.67% (33/36)***	27.19 ± 0.90*
				
Intact female	4	17	23.53% (4/17)	24.90 ± 1.99
Spayed female	13		76.47% (13/17)**	26.50 ± 1.02
				
Neutered before 6 months	11	53	20.75 (11/53)	32.39 ± 0.72
Neutered after 6 months	42		79.25% (42/53)**	25.22 ± 0.63*

** *p* < 0.01, *** *p* < 0.01 The percentage of data was analyzed using a two-sample proportion test with Minitab software (version 16.1) to determine if there were any significant differences between the two groups.

**Table 6.** Body weight and age dependence occurrence of cranial cruciate ligament rupture.

	NO (*n* = 53)	Occurrencee
BW		
Less than 10 kg	31	58.49% (31/53)
10~25 kg	19	35.85% (19/53)**
25 kg~	3	5.66% (3/53)***
Age		
Less than 3 year	2	3.77% (2/53)
3~5 year	12	22.64% (12/53) **
5~10 year	28	52.83% (28/53) ***
10 year~	11	20.75% (11/53) **

** *p* < 0.01, *** *p* < 0.01 The percentage of data was analyzed using a two-sample proportion test with Minitab software (version 16.1) to determine if there were any significant differences between the two groups.

The average age of the RCCL-diagnosed dogs was 7.04 ± 0.41 years, which was supported by previous reports [2,19]. A study showed that dogs between the ages of 9.0 and 11.9 years were 4.4 times more likely to be diagnosed with RCCL compared to dogs under 3 years old. This can occur due to heightened ligament degradation in elderly dogs in comparison to younger ones [2,6]. The occurrences in the less than 3 years, 3–5 years, 5–10 years, and over 10 years groups in this study were 3.77%, 22.64%, 52.83%, and 20.75%, respectively. These results are consistent with previous reports as the height occurrence was observed in the 5–10 years group, which was 14.01 and 2.33 times higher than that in the less than 3 years and 3–5 years groups. However, the comparatively lower occurrence of RCCL in the over 10 years group than in the 5–10 years group remains unclear.

## Conclusion

In conclusion, increased TPAs in neutered dogs are a risk factor for developing RCCL disease, especially in dogs neutered before 6 months of age. Cocker Spaniels and Maltese had a higher risk. Older age (between 5 and 10 years) was found as a risk associated with being diagnosed with CCL disease. Therefore, BW, age, sex, neutering status, and breed factors may be associated with RCCL in dogs. Importantly, lower pre-operative (≤ 25°) and post-operative TPA (≤ 10°) showed better prognoses in RCCL dogs. Therefore, pre- and postoperative TPA is an important prognostic factor, and postoperative TPA should be kept near 5° or <10° to get better clinical outcomes.

## List of Abbreviations

ECG, electrocardiogram; RCCL, cranial cruciate ligament rupture; TPA, Tibial plateau angle; TPLO, tibial plateau leveling osteotomy.
